# Successful Interventional Management for Pulmonary Arterial Injury Secondary to Pacemaker Implantation

**DOI:** 10.1155/2016/4340193

**Published:** 2016-11-02

**Authors:** Hiroyuki Tokue, Azusa Tokue, Hideo Morita, Yoshito Tsushima

**Affiliations:** ^1^Department of Diagnostic and Interventional Radiology, Gunma University Hospital, Gunma, Japan; ^2^Department of Radiology, Maebashi Red Cross Hospital, Maebashi, Gunma, Japan

## Abstract

Subclavian vein puncture is a relatively fast and safe technique to access the right heart for placement of pacemaker leads. Hemothorax related to injury of the pulmonary artery (PA) is a rare complication of subclavian vein access but can be life-threatening. We report a case of hemothorax occurring after subclavian vein puncture for pacemaker implantation. No cases of transcatheter arterial embolization for PA injury secondary to pacemaker implantation have been reported. Understanding of this rare complication after pacemaker implantation along with its specific clinical presentation may lead to early diagnosis and intervention.

## 1. Background

Pacemaker implantation is a routine procedure in modern cardiology. Although it is a safe procedure, one should not underestimate the possibility of complications. A case of accidental pulmonary arterial injury resulting in massive hemothorax after pacemaker implantation is presented.

## 2. Case Presentation

A 90-year-old man was admitted to our hospital to receive a permanent implanted pacemaker (VVI mode) to treat completed atrioventricular block. The patient had a medical history of percutaneous coronary intervention to the right coronary artery 10 years ago. He had a normal serum hemoglobin, platelets, red blood cell count, prothrombin time, and partial thromboplastin time. He was not taking any anticoagulant or antiplatelet medications.

**Figure 1:**
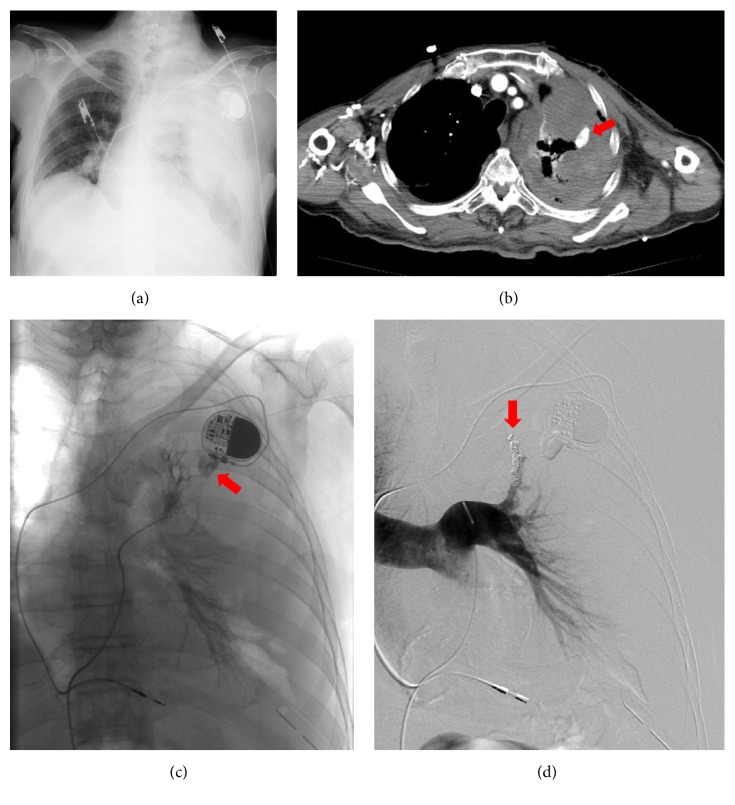
A 90-year-old man received a permanent implanted pacemaker. After the procedure, the patient's blood pressure steadily decreased. Left hemothorax and laceration of the pulmonary arterial branch of the left upper lobe were confirmed. (a) Chest radiograph showed a left hemothorax. (b) Contrast-enhanced computed tomography (CT) showed a confined cavity (pseudoaneurysm) of a branch of the upper lobe pulmonary artery (arrow). (c) Angiography demonstrated bleeding from the perforated pulmonary arterial branch of the left upper lobe (arrow). (d) Transcatheter arterial embolization (TAE) was performed using microcoils (arrow). Digital subtraction angiographic image taken after coil embolization discloses coils (arrows) in the upper lobe pulmonary artery and no further opacification of the pseudoaneurysm.

A decision to implant a pacemaker was made, and we used left subclavian vein puncture, which is our preferred technique for venous access. The operator had almost 20 years of experience in cardiac pacing. The infraclavicular approach based on anatomic landmarks was used without the use of ultrasonographic guidance. Left subclavian vein access was attempted with an 18-gauge needle, a tear-away introducer 9 Fr sheath, and a guidewire with a J-shaped soft tip. The fluoroscopic guidance for guidewire navigation was used. Venous access was hard, and multiple puncture trajectories and sites were attempted. With venous access, there was blood return. A guidewire was inserted, the needle was removed, and the dilator was advanced. However, the pacemaker leads were then inserted with considerable difficulty and were removed. Venous access was reattempted. Finally, access to the left subclavian vein and implantation of the pacemaker succeeded. There was no established evidence of arterial puncture or needle-air reflux suggesting parenchymal puncture. The patient was sedated and had no complaints. During the procedure, he had normal oximetry, perioperative electrical parameters were adequate, and the pacing system was implanted. The procedure ended without incident. However, in the hour after the procedure, the patient's blood pressure steadily decreased while his heart rate steadily increased. Hemodynamic deterioration did not respond to fluid resuscitation. A chest radiograph showed a left hemothorax (Figure 1(a)). A left hemothorax and pseudoaneurysm of the pulmonary arterial branch of the left upper lobe were confirmed on contrast-enhanced computed tomography (CT) (Figure 1(b)). Digital subtraction angiography (DSA) demonstrated the perforated pulmonary arterial branch of the left upper lobe bleeding into the left thoracic cavity (Figure 1(c)). The branch was quickly cannulated with a 5 Fr multipurpose catheter (Clinical Supply Co. Ltd., Gifu, Japan), and a Progreat 2.7 Fr coaxial microcatheter (Terumo Corp., Tokyo) was advanced near the bleeding point by using a Transend EX 0.014-in. microguidewire (Boston Scientific Corp., Watertown, MA, USA). We placed the microcatheter near the bleeding point, and transcatheter arterial embolization (TAE) was performed using three 5 × 2 mm Tornado coils (Cook Medical, Bloomington, IN, USA) and six 6 × 2 mm Tornado coils. After TAE, DSA did not demonstrate pseudoaneurysm (Figure 1(d)) and the patient became hemodynamically stable. He was discharged from the hospital without any major complications 30 days after TAE.

## 3. Discussion

Subclavian vein puncture is a relatively fast and safe technique to access the right heart for placement of pacemaker leads. It has been used since the beginning of the era of endocavitary pacemaker lead placement [1, 2] and has often been the preferred access technique for many operators [3]. A literature review identified similar cases. Complications such as alveolar hemorrhage, hemothorax, and hemoptysis are uncommon (<0.1%) in this setting [4, 5]. Moreover, there may be severely adverse outcomes in critically ill patients [2, 6]. However, the actual incidence of this complication is unknown, because cases are probably underreported.

To the best of our knowledge, there are no reports in the literature about TAE for pulmonary arterial injury after pacemaker implantation. The actual mechanism of pulmonary arterial injury due to subclavian vein puncture was not clear. In our case, we speculated that the guidewires were probably inserted into the pulmonary arterial branch of the left upper lobe. The dilatation may have created an aperture for perforation of the pulmonary arterial branch.

It may be difficult to recognize complications, as in our case. Therefore, signs of a complication should be promptly noted and treated as soon as possible. The typical clinical presentation is sudden hypotension after the procedure, and ultrasound, CT, or even a direct radiograph can confirm hemothorax [7–9]. We were able to treat our case with immediate TAE. If the injury cannot be treated by TAE, emergency thoracotomy is another option, and surgical intervention should be considered.

## 4. Conclusion

Inadvertent puncture of a branch of the left pulmonary artery (PA) led to a left hemothorax. Immediate TAE was performed and the bleeding site was quickly controlled. Understanding of this rare complication after pacemaker implantation with its specific clinical presentation may lead to early diagnosis and intervention.

## Abbreviation

PA: Pulmonary artery
CT: Computed tomography
TAE: Transcatheter arterial embolization
DSA: Digital subtraction angiography.

## Additional Points

*Availability of Data and Materials.* The authors do not wish to share the patient's data. They respect the patient's rights to privacy and to protect his identity. The authors presented all the necessary information about his case report in the manuscript. Raw data regarding their patient are managed strictly.
